# Early Renoprotective Effect of Ruxolitinib in a Rat Model of Diabetic Nephropathy

**DOI:** 10.3390/ph14070608

**Published:** 2021-06-24

**Authors:** Mohamed M. El-Kady, Reham A. Naggar, Maha Guimei, Iman M. Talaat, Olfat G. Shaker, Maha Saber-Ayad

**Affiliations:** 1 Department of Medical Pharmacology, Faculty of Medicine, Cairo University, Cairo 11559, Egypt; mahammad_elkady@cu.edu.eg; 2 Department of Pharmacology, Faculty of Pharmacy, Misr University for Science and Technology, Giza 12411, Egypt; pookiemust@gmail.com; 3 Department of Pathology, Faculty of Medicine, Alexandria University, Alexandria 21526, Egypt; guimeimaha@gmail.com (M.G.); italaat@sharjah.ac.ae (I.M.T.); 4 Department of Clinical Sciences, College of Medicine, University of Sharjah, Sharjah 27272, United Arab Emirates; 5 Research Institute for Medical and Health Sciences, University of Sharjah, Sharjah 27272, United Arab Emirates; 6 Department of Biochemistry and Molecular Biology, Faculty of Medicine Cairo University, Cairo 11559, Egypt

**Keywords:** diabetic nephropathy, ruxolitinib, JAK inhibitors, glomerulosclerosis, tubulointerstitial fibrosis, rat model, TGF-β1, NF-κB, TNF-α

## Abstract

Diabetic kidney disease (DKD) is still one of the unresolved major complications of diabetes mellitus, which leads ultimately to end-stage renal disease in both type 1 and type 2 diabetes patients. Available drugs that suppress the renin–angiotensin system have partially minimized the disease impact. Yet, there is an unmet need for new therapeutic interventions to protect the kidneys of diabetic patients. In DN, glomerular sclerosis and tubulointerstitial fibrosis are mediated through several pathways, of which JAK/STAT is a key one. The current study explored the potential renoprotective effect of the JAK1/JAK2 inhibitor ruxolitinib (at doses of 0.44, 2.2, and 4.4 mg·kg^−1^) compared to that of enalapril at a dose of 10 mg·kg^−1^, in a rat model of streptozotocin-induced diabetes mellitus over 8 weeks. The effect of ruxolitinib was assessed by determining urinary albumin/creatinine ratio, serum level of cystatin, and levels of TGF-β1, NF-κB, and TNF-α in renal tissue homogenates by biochemical assays, the glomerular sclerosis and tubulointerstitial fibrosis scores by histological analysis, and fibronectin, TGF-β1, and Vimentin levels by immunohistochemical staining with the respective antibodies. Our results revealed a significant early favorable effect of a two-week ruxolitinib treatment on the renal function, supported by a decline in the proinflammatory biomarkers of DKD. This pre-clinical study suggests that the renoprotective effect of ruxolitinib in the long term should be investigated in animals, as this drug may prove to be a potential option for the treatment of diabetic kidney disease.

## 1. Introduction

Diabetes mellitus is the major cause of end-stage renal disease (ESRD) all over the world. The global percentage of diabetes patients with ESRD has increased to around 30% [1]. Approximately 47% of ESRD cases in the United States are due to diabetic nephropathy [2]. A good control of diabetes positively impacts the natural course of the disease, as shown by a decline in the rate of worsening of albuminuria and of plasma creatinine levels; nonetheless, renal dialysis or transplantation remains, unfortunately, the ultimate outcome [3].

The mechanism of ESRD in diabetes involves several proinflammatory and profibrotic pathways that are activated secondary to hyperglycemia. This causes damage to the filtration barrier of the glomerulus, loss of podocytes, expansion of the mesangial matrix, tubulointerstitial fibrosis, and decreased glomerular filtration rate [4]. Loss of the barrier integrity of the renal glomerulus promotes the development of albuminuria. The latter further adds to the toll of tubulointerstitial fibrosis [4,5]. Furthermore, the levels of angiotensin II (Ang II) are 1000-fold higher in the renal interstitial tissue than in the plasma of patients with diabetes, which, in turn, contributes to a remarkable damaging effect [6]. Intrarenal Ang II also induces inflammation and leads to increased intraglomerular pressure and glomerular permeability, culminating in albuminuria [7].

A growing body of evidence has shown that chronic inflammation of the kidney is a crucial contributing factor in ongoing diabetic kidney disease (DKD). The transcription factor nuclear factor kappa-light-chain-enhancer of activated B cells (NF-κB), a well-known organizer of many inflammatory processes, is upregulated in DKD by the increased level of tumor necrosis factor (TNF) and underlying oxidative stress. Also, NF-κB has been linked to the renal recruitment of inflammatory cells, together with the loss of proteins in urine [8]. In addition, the hyperglycemic state induces the expression of transforming growth factor-β1 (TGF-β1) in podocytes and proximal tubular cells. TGF-β1 further induces the synthesis of laminin, fibronectin, and type-4 collagen in the glomeruli, causing renal scarring [9].

Several intricate processes are initiated and activated by hyperglycemia to induce tubular fibrosis in DN, with a myriad of cells (including interstitial fibroblasts, endothelial cells, tubular cells, and pericytes) and matrix proteins produced by the activated myofibroblasts (such as fibronectin, laminin, and collagen I, III, and IV). Fibronectin and collagen IV are major ECM proteins that serve as a scaffold for the deposition of other proteins, such as collagen type I and III, leading to interstitial fibrosis [10]. The details of DN pathogenesis have been described by Loeffler and Wolf [11].

In the nephron, N-acetyl-seryl-aspartyl-lysyl-proline (Ac-SDKP) is released from thymosin β4. The angiotensin-converting enzyme (ACE) N-domain converts the natural substrate N-acetyl-seryl-aspartyl-lysyl-proline (Ac-SDKP) into inactive fragments [12]. The thymosin β4–Ac-SDKP axis prevents kidney fibrosis under normal conditions [13] and can reduce fibrosis during kidney injury [14], adding to the renoprotective effect of ACE inhibitors. Furthermore, the ACE inhibitors alone or combined with Ac-SDKP inhibits the renal overexpression of the enzyme dipeptidyl peptidase-4 (DPP-4) and the activation of TGF-β signaling via the anti-fibrotic microRNAs miR-29s and miR-let-7s, leading to decreased endothelial– and epithelial–mesenchymal transition, as well as to a reduction in renal ECM deposition [15,16].

ACE inhibitors and the angiotensin receptor blockers (ARBs) have been established as a standard treatment for decades, (Bergamo Nephrologic Diabetes Complication Trial (BENEDICT) using the ACE inhibitor, trandolapril [17], Reduction of Endpoints in NIDDM with the Ang II Antagonist Losartan (RENAAL) [18], DETAIL (Diabetics Exposed to Telmisartan and Enalapril) using a drug combination [19]). Enalapril has been investigated in many clinical trials, and its antiproteinuric effect has been demonstrated a long time ago, independent of its effect on blood pressure [20].

Many antidiabetic medications and other drugs used in diabetes showed a promising effect in the treatment of DN in experimental animal models. Medications targeting the vasculature include glucagon-like peptide 1 (GLP-1) agonists and sodium–glucose transport protein 2 (SGLT2) inhibitors [21]. A recent study showed that the sodium–glucose co-transporter-2 (SGLT2) inhibitor empagliflozin and the dipeptidyl peptidase-4 (DPP4) inhibitor linagliptin can reactivate autophagy in the glomeruli of a mouse model of type 2 DM, adding to the mechanisms of their renoprotective effect [22].

Clinical trials on relatively newer anti-diabetic medications involved DPP-4 inhibitors [23] and SGLT-2 inhibitors [CANTATA-SU (Canagliflozin Treatment and Trial Analysis versus Sulphonylurea). Interestingly, the SGLT-2 inhibitor dapagliflozin was also reported to rectify the glucose-induced metabolic shift in proximal tubular renal cells via inhibiting HIF-1α [24].

In addition, a myriad of investigational molecules have been explored, e.g., the endogenous antifibrotic peptide AcSDKP that showed a renoprotective effect in mice via suppressing endothelial–mesenchymal transition and restoring the expression of the let-7 microRNA family [25]. A recently suggested approach is through the inhibition of aerobic glycolysis-mediated epithelial–mesenchymal transition (EMT), thus suppressing renal interstitial fibroblast activation and renal fibrosis [26].

The management of hypertension and the suppression of the renin–angiotensin–aldosterone system (RAAS) serve to decrease the severity of albuminuria in patients with diabetes mellitus [19,27]. However, there is still a significant risk of DKD progression, even under RAAS inhibitor therapies [28]. Therefore, other strategies of renoprotection in diabetes patients were suggested, including those mitigating inflammation, fibrosis, and oxidative stress [29,30]. However, the long-term follow-up of diabetic patients has shown the progression of renal disease despite the use of these drugs in several clinical trials [31,32].

Recently, the Janus kinase–signal transducer and activator of transcription (JAK-STAT) pathway has been strongly implicated in the pathogenesis of metabolic diseases including diabetes [33]. The expression of JAK–STAT family members was found to be considerably elevated in human diabetic kidney tissues [34,35]. Selective inhibition of JAK–STAT signaling by orally bioavailable small molecules, exemplified by ruxolitinib, tofacitinib, and baricitinib, has been accepted for clinical use in various autoimmune and inflammatory diseases [36,37].

In this study, we aimed to investigate the potential renoprotective effect of ruxolitinib, a JAK1–JAK2 inhibitor, in a streptozotocin-induced diabetic rat model. By comparing biochemical, renal tissue proinflammatory biomarkers, and histopathological changes, our findings show a favorable effect of ruxolitinib in the rat model of DKD.

## 2. Results

### 2.1. Effect of Ruxolitinib on Serum Cystatin, Albumin Excretion Ratio, Urinary Albumin, and Urinary Creatinine Levels

**Figure 1:**
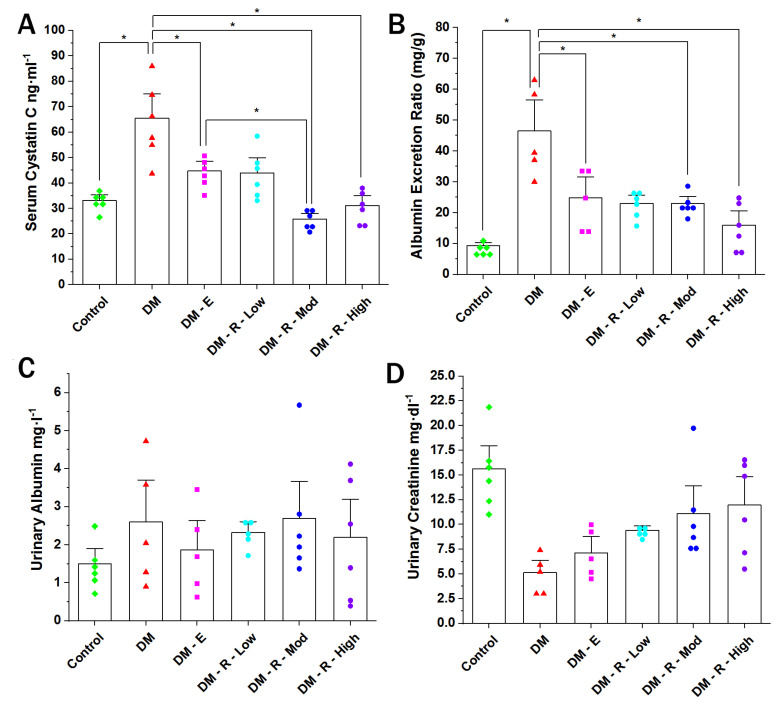
Effect of ruxolitinib on serum cystatin (**A**), albumin excretion ratio (**B**), urinary albumin (**C**), and urinary creatinine levels (**D**). (**A**) Cystatin C level in the sera of normal, diabetic treated, and non-treated male Wistar rats. (B) Bar graph showing urinary albumin excretion of normal (control), diabetic treated, and non-treated male Wistar rats (*n* = 6). Data are represented as individual values for each mouse (dots). Statistical significance is shown by asterisks (* *p* < 0.05). The difference among groups was assessed by the Kruskal–Wallis test followed by the Mann–Whitney U test for post hoc group binary comparisons. DM, diabetic non-treated group, DM-E, diabetic enalapril-treated group (10 mg·kg^−1^), DM-R-Low, diabetic ruxolitinib-treated low-dose group (0.44 mg·kg^−1^), DM-R-Mod, diabetic ruxolitinib-treated moderate-dose group (2.2 mg·kg^−1^), DM-R-High, diabetic ruxolitinib-treated high-dose group (4.4 mg·kg^−1^).

A ten-week period of diabetes in rats nearly doubled the serum level of cystatin (from 33.11 ± 1.53 to 65.53 ± 6.36 ng·mL^−1^) (Figure 1A). The cystatin level in the serum of diabetic non-treated animals was significantly elevated compared to that of the normal non-diabetic group. Enalapril (10 mg·Kg^−1^, orally) significantly decreased the serum cystatin level in comparison to the diabetic non-treated group from 65.53 ± 6.36 to 44.76 ± 3.95 ng·mL^−1^. Treatment with moderate and high doses of ruxolitinib (2.2 & 4.4 mg·kg^−1^, respectively) significantly decreased the cystatin level in comparison to the diabetic non-treated group. Also, a moderate dose of ruxolitinib significantly decreased the serum cystatin level compared to that in the enalapril-treated group, while the high dose showed no statistical distinction with respect to the enalapril-treated group.

The ACR of diabetic non-treated animals was significantly elevated compared to that of the normal non-diabetic group (from 9.25 ± 0.75 to 46.48 ± 6.12 mg/g, Figure 1B). Enalapril (10 mg·Kg^−1^, orally) significantly decreased the ACR in comparison to the diabetic non-treated group, from 46.48 ± 6.12 to 24.78 ± 4.17 mg/g. Treatment with moderate and high doses of ruxolitinib (2.2 and 4.4 mg·kg^−1^, respectively) significantly decreased the ACR in comparison to the diabetic non-treated group. Group comparison of the effects of enalapril and moderate and high doses of ruxolitinib revealed no statistical difference, denoting equal efficacy.

### 2.2. Effects of Ruxolitinib on Kidney NF-κB and TGF-β1 Levels

**Figure 2:**
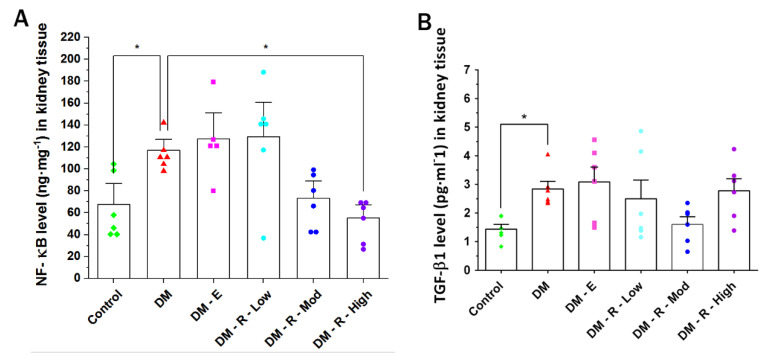
Effect of ruxolitinib on kidney NF-κB (**A**) and TGF- β1 (**B**) levels. (**A**) NF-κB level in kidney tissue homogenates of normal (control) rats and (**B**)TGF-β1 level in kidney tissue homogenates of normal (control), diabetic treated, and diabetic non-treated male Wistar rats. DM, diabetic non-treated group, DM-E, diabetic enalapril-treated group (10 mg·kg^−1^), DM-R-Low, diabetic ruxolitinib-treated low-dose group (0.44 mg·kg^−1^), DM-R-Mod; diabetic ruxolitinib-treated moderate-dose group (2.2 mg·kg^−1^), DM-R-High, diabetic ruxolitinib-treated high-dose group (4.4 mg·kg^−1^). Each column represents the mean (±S.E.M.) for 6 male Wistar rats. Data are represented as individual values for each mouse (dots). Statistical significance is shown by asterisks (* *p* < 0.05). The difference among groups was assessed by the Kruskal–Wallis test followed by the Mann-Whitney U test for post hoc group binary comparisons.

The NF-κB level in kidney tissue homogenates from diabetic non-treated animals was significantly elevated compared to that from the control non-diabetic group (from 67.77 ± 12.59 to 116.85 ± 6.71 ng·mL^−1^) (Figure 2A). On the other hand, high-dose ruxolitinib (4.4 mg·kg^−1^, orally) significantly decreased the renal NF-κB level, in contrast with what observed in the diabetic non-treated group (from 67.77 ± 12.59 to 55.20 ± 8.01 ng·mL^−1^).

The TGF-β1 level in kidney tissue homogenates of diabetic non-treated animals was significantly elevated compared to that in the control non-diabetic group (from 1.41 ± 0.17 to 2.84 ± 0.26 pg·mL^−1^) (Figure 2B). Unexpectedly, enalapril (10 mg·Kg^−1^, orally) significantly elevated renal TGF-β1 level, in contrast with what observed in the diabetic non-treated group, from 2.84 ± 0.26 to 5.75 ± 0.23 pg·mL^−1^. No statistical significance was shown for differences between groups treated with different doses of ruxolitinib and the diabetic non-treated group.

### 2.3. Effect of Ruxolitinib on Kidney TNF-α and IL-1β Levels

**Figure 3:**
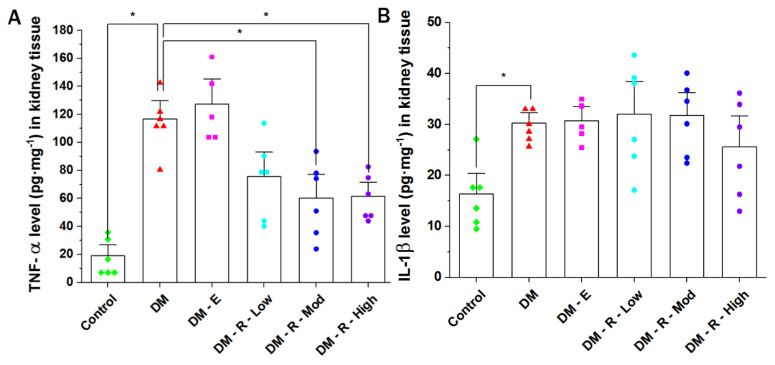
Effect of ruxolitinib on the kidney (**A**) TNF-α and (**B**) IL-1β levels. (**A**) TNF-α level in kidney tissue homogenates of normal (control) rats and (**B**) IL-1β level in kidney tissue homogenates of normal (control) and diabetic treated and non-treated male Wistar rats. DM, diabetic non-treated group, DM-E, diabetic enalapril-treated group (10 mg·kg^−1^), DM-R-Low, diabetic ruxolitinib-treated low-dose group (0.44 mg·kg^−1^), DM-R-Mod, diabetic ruxolitinib-treated moderate-dose group (2.2 mg·kg^−1^), DM-R-High, diabetic ruxolitinib-treated high-dose group (4.4 mg·kg^−1^). Each column represents the mean (±S.E.M.) for 6 male Wistar rats. Data are represented as individual values for each mouse (dots). Statistical significance is shown by asterisks (* *p* < 0.05). The difference among groups was assessed by the Kruskal–Wallis test followed by the Mann–Whitney U test for post hoc group binary comparisons.

The TNF-α level in kidney tissue homogenates of diabetic non-treated animals was significantly elevated compared to that in the control non-diabetic group (from 19.11 ± 5.19 to 116.62 ± 8.84 pg·mL^−1^) (Figure 3A), while moderate-dose ruxolitinib (2.2 mg·kg^−1^, orally) significantly decreased the renal TNF-α level in comparison with the diabetic non-treated group, from 116.62 ± 8.84 to 60.23 ± 11.26 pg·mL^−1^. High-dose ruxolitinib (4.4 mg·kg^−1^, orally) also showed a significant decrease in renal TNF-α level in comparison with the levels in the diabetic non-treated group, from 116.62 ± 8.84 to 61.64 ± 6.57 pg·mL^−1^

The IL-1β level in kidney tissue homogenates of diabetic non-treated animals was significantly elevated compared to the level in the control non-diabetic group (from 16.35 ± 2.71 to 30.29 ± 1.32 pg·mL^−1^) (Figure 3B). No statisticallysignificant difference was shown when comparing the control with the enalapril group or groups treated with different doses of ruxolitinib with the diabetic non-treated group.

### 2.4. Effects of Ruxolitinib on Renal Histological Findings in Streptozotocin-Induced Diabetic Rats

Histopathological evaluation of the kidneys from STZ-induced diabetic rats revealed evidence of glomerular injury in the form of increased basement membrane thickening, increased mesangial matrix, and mild focal segmental sclerosis. The tubulointerstitial injury was also confirmed by the presence of foci of tubular dilatation, tubular atrophy, interstitial inflammation, and less evident areas of fibrosis.

**Figure 4:**
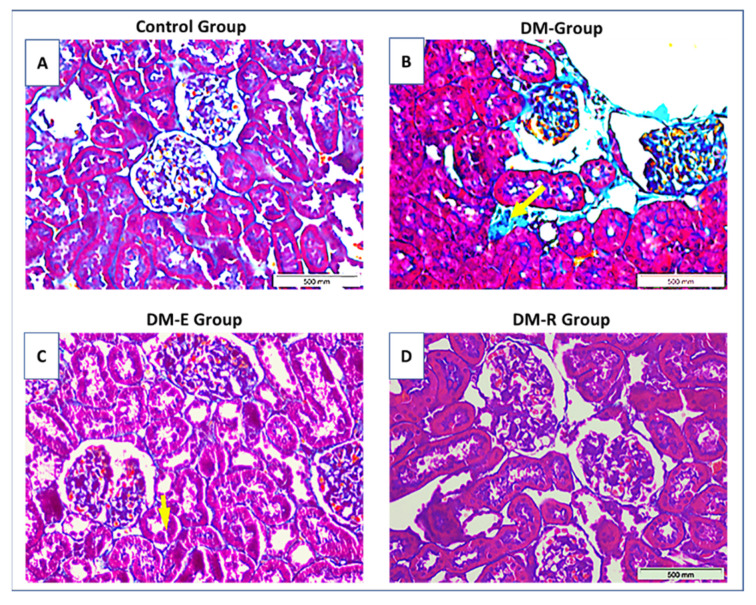
Light-microscopic findings in the study groups. Representative pictures of Masson Trichrome-stained tissue sections from (**A**–**D**) Ctrl group (**A**), DM group (**B**), enalapril-treated group DM-E (**C**), and ruxolitinib-treated group DM-R (**D**). A. Control group showing normal glomerular and tubular structures. B. Glomeruli in the DM group showing increased periglomerular fibrosis (stained in blue) as well as interstitial fibrosis (arrow). C. DM-E group showing improvement of both glomerular and interstitial fibrosis. D. DM-R group showing improvement of both glomerular and tubular changes. Note that glomerular diabetic changes are less evident in the DM-R group than in the DM-E group. Ctrl: control, DM: untreated group, DM-E: enalapril-treated group, DM-R: ruxolitinib-treated group. Original magnifications ×400.

The mean glomerular sclerosis score (GSS) in the DM group was 2.7/4, significantly higher than that in the control group, which was evaluated as 0/4, (*p* < 0.001). However, treatment with enalapril as well as with medium and high doses of ruxolitinib resulted in a decrease in the mean GSS score in DM-E, DM-R-Mod, and DM-R-high groups, to 1.2/4, 1.0/4, and 1.7/4, respectively, thus proving a significant reduction in the GSS in all three groups (*p* = 0.04, 0.02 and 0.14, Mann–Whitney U test, respectively) compared to the untreated group (DM) (Figure 4A–D).

**Figure 5:**
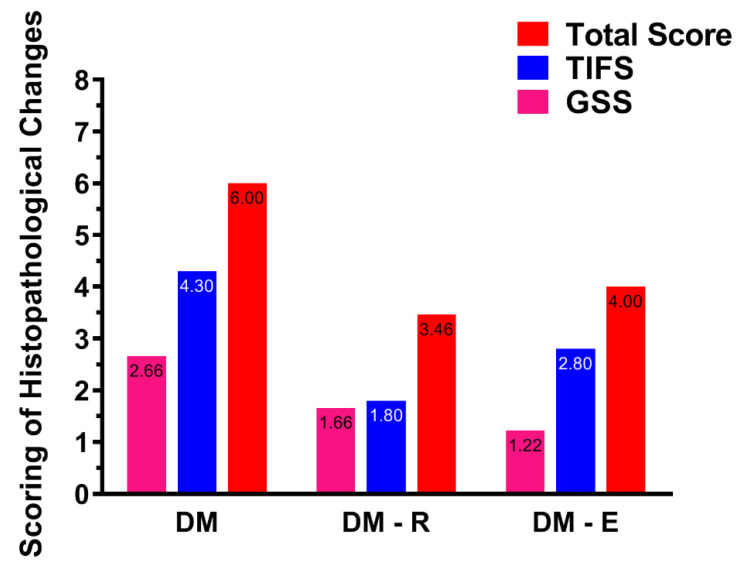
The effect of treatments with enalapril and ruxolitinib on GSS and TIFS in the studied groups. Treatment with ruxolitinib resulted in an almost equal reduction of GSS and TIFS (*p* = 0.14 and 0.03, respectively). The effect of enalapril treatment was more significant on GSS than on TIFS (*p* = 0.04 and 0.02, respectively). DM, diabetic non-treated group, DM-E, diabetic enalapril-treated group (10 mg·kg^−1^), DM-R, diabetic ruxolitinib-treated group (moderate dose of 2.2 mg·kg^−1^).

The mean tubulointerstitial fibrosis score (TIFS) in the DM group was 4.3/5. Treatment with enalapril as well as with medium and high doses of ruxolitinib also resulted in a significant reduction of TIFS to 2.8/5, 1.0/5, and 1.8/5, respectively (*p* = 0.02, 0.03, and 0.03, Mann–Whitney U test), (Figure 5).

**Figure 6:**
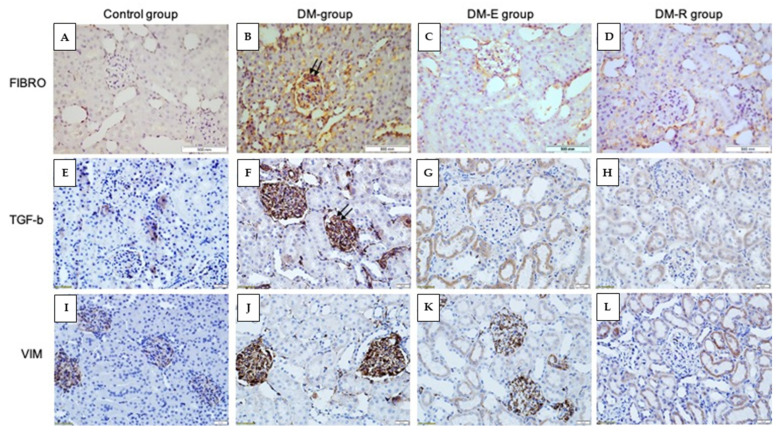
Immunohistochemistry analysis of fibronectin (**A**–**D**), TGF-β1 (**E**–**H**), and vimentin (**I**–**L**) expression in the studied groups. Representative pictures after immunostaining for fibronectin, TGF-β1, and vimentin in the Ctrl group (**A**,**E**,**I**), DM group (**B**,**F**,**J**), enalapril group (**C**,**G**,**K**), and DM-R group (**D**,**H**,**L**). Note that glomeruli in the DM group show evidence of focal glomerular sclerosis, as indicated by an increased percentage of the stained area (double arrows), whereas in both DM-E and DM-R groups, glomeruli and tubules do not show evidence of injury and look almost similar to glomeruli in the Ctrl group. DM, diabetic non-treated group, DM-E, diabetic enalapril-treated group (10 mg·kg^−1^), DM-R, diabetic ruxolitinib-treated group (moderate dose of 2.2 mg·kg^−1^). FIBRO; fibronectin, VIM; vimentin. Original magnifications ×400.

Immunohistochemical evaluation of the TGF-β1-, vimentin-, and fibronectin-stained sections from all groups also showed a significant reduction of the percentage of glomerular tuft area occupied by stained matrix (Figure 6A–L).

## 3. Discussion

The incidence of DKD is continuing to increase because of the type-2 diabetes epidemic. However, there is some improvement in the rates of progressive DKD [37]. It is undoubtful that angiotensin-converting enzyme inhibitor the angiotensin receptor blocker therapies, as well as blood pressure control, have effectively slowed down DKD progression to ESRD, but further powerful pharmacological interventions are required.

In a transcriptomics study, Brosius et al. found that the expression of multiple JAK–STAT family members was significantly upregulated in kidney tissues from humans with DKD [34]. Upon ligand binding, JAK membrane receptors dimerize, recruit pairs of JAK proteins to the intracellular receptor domains, and activate them via autophosphorylation. Consequently, activated JAKs phosphorylate STAT proteins, which translocate to the nucleus to induce target gene transcription of a variety of cytokines, adhesion molecules, growth factors, extracellular matrix proteins, pro-oxidant enzymes, and scavenger receptors, leading to several pathologies related to DKD, including inflammation, oxidative stress, lipotoxicity and fibrosis [38,39].

In this study, we developed a robust model for DKD, as previously described [40]. To compare RAAS versus JAK inhibition, we used an ACE inhibitor, rather than an ARB, as there is a class effect of RAAS inhibition. ARB may show an inconsistent effect on blood pressure and glomerular filtration rate (GFR) in several animal models [41]. As an example, an in vivo study showed an increased GFR upon acute losartan treatment in rats [42]; however, such effect was not found in another study using eprosartan [43].

Our results showed a significant reduction of the serum cystatin level upon treatment with ruxolitinib compared to treatment with enalapril. Serum cystatin C was shown previously to be a useful marker of early renal impairment in type 2 diabetic patients. The cystatin level reflects both a decrease in GFR and an elevated ACR [44]. As expected, the cystatin level in our study correlated with ACR changes. Our results showed that ruxolitinib at moderate and high doses induced a reduction in ACR that was equal to that detected in the enalapril group, denoting an equal efficacy of both medications in reducing albuminuria. Ruxolitinib is a JAK1/2 inhibitor, as DN pathogenesis involves JAK2 [45]. Notably, various JAK inhibitors vary in their binding affinity, e.g., tofacitinib has a higher binding affinity to JAK3 compared to JAK1/2 [46].

In the current study, different doses of ruxolitinib showed a significant reduction of inflammatory biomarkers, measured in renal homogenates. In DKD, hyperglycemia stimulates the overexpression of inflammatory mediators by injured glomerular and tubular cells. This results in renal damage through several mechanisms, i.e., immune cell infiltration, mesangial proliferation, and ultimately, podocyte/tubular damage [47]. These inflammatory mediators upregulate a myriad of signaling pathways, including NF-κB, JAK/STAT, and TGFβ/Smad, leading to extracellular matrix deposition and myofibroblast proliferation [48]. In our study, TGF-β1 was significantly reduced by ruxolitinib treatment. The enalapril-treated group showed a significant reduction of both GSS and TIFS compared to the DM-untreated group, in contrast to a slight increase in inflammatory markers measured by ELISA. The insignificant increase in the level of inflammatory markers may be due to increased plasma renin, which is expected upon enalapril treatment [49]. Some studies also showed that renin and TGF-β are coregulated, and both increase under the effect of ACE inhibition. Other studies showed that renin can directly increase TGF-β in a dose-dependent and time-dependent manner [50]. Due to the short duration of treatment (only 2 weeks), our data did report the expected renoprotective effect of enalapril treatment, which is likely to occur with a longer duration of treatment.

IL-1β was significantly elevated in the diabetic non-treated group compared to the control. A high dose of ruxolitinib showed a trend toward a reduction of IL-1β, though statistically insignificant. IL-1β is unique among other cytokines, having gasdermin D-dependent and -independent secretion [51]. This may explain its long duration of recycling in the immune cells and the lack of correlation with TNF-α in the current study, although both are secreted from macrophages in DKD along with other inflammatory cytokines (e.g., TNF-α), reactive oxygen species, chemokines, complement factors, and metalloproteinases [52].

In the current study, ruxolitinib at moderate and high doses significantly decreased renal TNF-α levels. Similarly, ruxolitinib treatment significantly decreased renal NF-κB levels. In DKD, hyperglycemia-mediated PKCβ and PKCδ activation in the renal cortex leads to activation of NF-κB and release of TNF-α by endothelial and mesangial cells [53]. Previous studies showed that PKCβ and PKCδ gene deletion decreased renal hypertrophy, apoptosis of podocytes, fibrosis, proteinuria, and endothelial dysfunction in diabetic mice [54,55].

The immunohistochemical assessment of renal samples provided results in correlation with those of renal tissue cytokine assays, as evaluated by the glomerular sclerosis score and the tubulointerstitial fibrosis score. We used fibronectin and vimentin as markers of pathological changes occurring in DKD. One of the major pathological characteristics of diabetic renal fibrosis is the accumulation of glomerular extracellular matrix, of which fibronectin is an important component, whose levels reflect the severity of DKD in the rat model [56]. Vimentin is a mesenchymal marker that is expressed in the interstitium of the kidney during the process of regeneration [57]. In addition, vimentin expression is implicated in EMT-related kidney fibrosis [58]. The use of the Glomerular Sclerosis Score is well-recognized for evaluating histopathological changes in DKD [59]. Along with sclerosis of the glomeruli, tubulointerstitial fibrosis is another hallmark of DKD [60]. We used the tubulointerstitial scoring system previously described by Kuno et al. [61].

Myofibroblasts are the direct effectors in the pathogenesis of interstitial fibrosis. A myriad of myofibroblast progenitors exist, e.g., bone marrow-derived cells, resident interstitial fibroblasts, perivascular mesenchymal stem cells, and endothelial cells that transform into myofibroblasts through epithelial-to-mesenchymal transition (EMT) and endothelial-to-mesenchymal transition (EndMT), [62].

As a part of chronic inflammation, endothelial cells are continuously activated by inflammatory stimuli, including IL-6, TNF-α, and IL-1β, resulting in endothelial dysfunction and the progression of fibrosis [63].

Inflammation-induced EndMT, similar to EMT, is mediated by two key signaling pathways: TGFβ and non-TGFβ pathways [64]. TGFβ increases the expression of transcription factor, e.g., snail, slug, and zinc finger E-box-binding homeobox 1 (ZEB1), which, in turn, upregulate the expression of several mesenchymal markers, e.g., alpha-smooth muscle actin, calponin, vimentin, fibronectin, N-cadherin, matrix metalloprotein (MMP)-2, and MMP-9 [65].

The JAK/STAT pathway is highly activated in DN and is involved in the progression of the disease. Such overactivation is a consequence of the prevalent status of inflammation, oxidative stress, lipid accumulation, lipotoxicity, and fibrosis associated with DN. The JAK/STAT pathway cross-talks with several signaling pathways, e.g., MAPK/ERK and PI3K/Akt/mTOR, in the intricate pathogenesis of DN [66]. JAK/STAT pathway activation ultimately leads to gene overexpression of different cytokines, adhesion molecules, transcription factors, and growth factors [37]. Furthermore, activation of Jak/Stat was previously reported to mediate downstream Ang II signaling and to induce the expression of TGF-β [34]. Accordingly, JAK inhibitors, e.g., ruxolitinib, are likely to exert a significant amelioration of such effect. In a transcriptomic analysis of renal samples from patients with DN, several upregulated pathways involved JAK2, namely, dendritic cell maturation, interferon signaling, CTLA4 signaling, acute-phase response signaling, PDGF signaling, and ephrin receptor signaling (the largest subfamily of receptor tyrosine kinases), [67]. Notably, JAK2 is a key link among different inflammatory pathways in DN. A highlight of the functional protein network of JAK2 shows its partner molecules that link key inflammatory pathways [68], (Supplementary Figure S1, and Supplementary Table S1).

The results of phase II clinical trials on baricitinib (another JAK1/JAK2 inhibitor) indicated reduced urinary ACR by 40% over 24 weeks in patients with Type 2 diabetes and DKD. The authors stated that further research is required to determine if baricitinib reduces DKD progression. Our study is a trial to explore the effect of JAK inhibition on histopathological changes in a diabetic kidney model and on the production of different cytokines within the renal tissue. However, this study had several limitations, including a small sample that led to the variability of the UACR and inflammatory biomarkers’ levels, lack of diversity, and low power than initially planned. Serum creatinine increased in the baricitinib groups, with small reductions in creatinine-based eGFR but no change in cystatin C-based eGFR. The authors justify the discrepancy by the baricitinib inhibition of renal tubular creatinine secretion [69]. Further research should be done to experimentally and clinically validate the results of this study.

Our study showed that the effect of the JAK inhibitor ruxolitinib was equal to that of enalapril in the DKD rat model examined and was greater with respect to some inflammatory tissue biomarkers A limitation of our study is that it did not assess the effect of combined ruxolitinib and ACE inhibitor therapy. Further research is needed to evaluate the effect of the combination of different doses of both medications in DKD, experimentally and through clinical trials.

Ruxolitinib shares 82.5% structural similarity with baricitinib. Noteworthy, baricitinib uptake by different tissues does not depend on organic cation transporters. There are significant differences between the two JAK inhibitors (also considered tyrosine kinase inhibitors) [70]. Ruxolitinib is metabolized by CYP3A4 and CYP2C19, whereas baricitinib metabolisms is CYP-independent, and the drug is excreted unchanged in the urine. Both medications are used for the treatment of a myriad of immune-mediated diseases [71]. Further studies on both medications should be carried out to validate their roles in experimental models of KDK as well as in clinical trials.

## 4. Materials and Methods

### 4.1. Animals

Experiments were carried out on male albino Wistar rats weighing 180–220 g that were housed and placed under a 12 h light/dark cycle in a temperature-controlled room (22 ± 2 °C). Animals had free access to food and water throughout the experiments. All experiments follow the guidelines of the local committee of Ethics on Animal Experimentation, Cairo University.

### 4.2. Model Induction

Diabetes was induced in overnight fasted rats by a single intraperitoneal (i.p.) injection of a freshly prepared streptozotocin (STZ) solution [55 mg/kg body weight (b/w)] dissolved in 0.1 M citrate buffer (pH = 4.5) in a volume of 1 mL/kg. After 72 h from STZ injection, plasma glucose levels were estimated with a standard glucometer from rat tail blood, and rats with fasting glucose levels greater than 300 mg/dL were considered diabetic and used for this experimental study. Diabetic rats were maintained on suboptimal doses of long-acting insulin (2 U insulin glargine) every other day to prevent ketoacidosis and weight loss [40].

### 4.3. Animal Groups and Treatment Protocol

The animals were randomly allocated to the following groups (6 animals each). The negative control group was represented by the normal non-diabetic group (control group) and a diabetic non-treated group (DM); whereas a diabetic enalapril-treated (DM-E) was used as a positive control. Ruxolitinib, the tested drug, was given to three diabetic groups: low-dose group (DM-R-Low), moderate-dose group (DM-R-Mod), and high-dose group (DM-R-High). Eight weeks after induction of diabetes, treatment was started with daily single dosing by gavage for 2 weeks, as follows. The DMgroup received saline, while the DM-E group was given enalapril (10 mg/kg) dissolved in saline (10 mL/kg) [72]. The diabetic ruxolitinib-treated groups received 0.44 mg/kg (DM-R-Low), 2.2 mg/kg (DM-R-Mod), and 4.4 mg/kg (DM-R-High). Ruxolitinib was dissolved in saline (10 mL/kg). Dosage of ruxolitinib was based on extrapolating the wide range of the daily therapeutic human doses (5 mg, 25 mg, 50 mg) [73,74] to rat species [75].

### 4.4. Drugs and Chemicals

The following drugs and chemicals were used: ruxolitinib (Medkoo Biosciences, Morrisville, NC, USA), enalapril maleate (Ezapril, Multi-Apex Pharma, Cairo, Egypt), insulin glargine (Lantus^®^ Solostar^®^ Sanofi Aventis), ketamine (Rotexmedica GmbH, Trittau, Germany), xylazine (xylaject 2%; Adwia, Cairo, Egypt), and strepto-zotocin (TOKU-E, Bellingham, WA, USA).

### 4.5. Collecting Biospecimens

At the end of the study, all animals were placed individually in metabolic cages on the day before sacrifice for collecting urine excreted over 24 h. The collected urine was stored at −20 °C for later measurement of albumin and creatinine concentrations. After collecting urine, the rats were anesthetized with an intraperitoneal injection of ketamine and xylazine (50 mg/kg and 10 mg/kg, respectively) [76], and 0.2 mL of blood was withdrawn from the tail veins of each animal.

The abdominal cavity was exposed by a midline incision. Animals were killed by exsanguinating blood through traversing the abdominal aorta, and both kidneys were immediately harvested. The right kidney was fixed in 10% formalin for 24 h before embedding in paraffin for histopathological analysis, and the left one was stored immediately at −80 °C until use.

### 4.6. Biochemical Assessment

Urinary albumin and creatinine concentrations were measured quantitatively using a spectrophotometer with the aid of commercial diagnostic reagents (supplied from Biosystems, Spain). The albumin creatinine ratio (ACR) was calculated as follows: urine albumin (mg/dL) ×1000)/urine creatinine (mg/dL). The urine ACR closely approximates the gold standard, 24 h urine albumin excretion mg/24 h while accounting for differences in urine volume [77].

Blood samples were allowed to coagulate at room temperature for one hour and then centrifuged at 500× *g* for 20 min. The separated serum was used to determine cystatin C levels, by applying the quantitative sandwich enzyme immunoassay method with a commercial Elisa kit (Sunlong Biotech, Hangzhou, China) [78].

Each frozen kidney was sectioned, comprising the cortex and medulla, to yield 100 mg of renal tissue. Subsequently, the reduced tissue was homogenized in 1 mL of ice-cold potassium phosphate buffer using a rotor–stator homogenizer. The resulting kidney homogenate was centrifuged, and the supernatant was used for the evaluation of the levels of cell signaling cytokines, namely, interleukin 1 beta (IL-1β), tumor necrosis factor-alpha (TNF-α), transforming growth factor-beta 1(TGF-β1), and nuclear factor kappa B (NF-κB) using specific ELISA kits (Abbkine Scientific, Wuhan, China).

### 4.7. Histopathological Evaluation of Renal Changes

Formalin-fixed paraffin-embedded (FFPE) kidney specimens from all the studied groups were sliced and stained with hematoxylin and eosin (H&E), Periodic Acid–Schiff (PAS) and Masson’s trichrome were used for staining, and the specimens were evaluated too determine the glomerulosclerosis and tubulointerstitial fibrosis scores.

The extent of glomerular damage in each rat was assessed by examining 50 glomeruli. A glomerulosclerosis score (GSS) was then calculated based on the number of glomeruli showing sclerotic changes out of the 50 examined glomeruli. The assessment was performed on PAS- and trichrome-stained sections using a semiquantitative score from 0 to 4: 0, no sclerosis; 1, sclerosis of up to 25% of glomeruli; 2, sclerosis in 25% to 50% of glomeruli; 3, sclerosis from 50% to 75% of glomeruli; 4, sclerosis of more than 75% of glomeruli [79].

Tubulointerstitial damage was defined as the presence of tubular dilatation, interstitial inflammation, and fibrosis. A tubulointerstitial fibrosis score (TIFS) was semi-quantitatively calculated based on the number of fields exhibiting morphological evidence of tubular damage out of 10 examined fields (magnification ×200). Scores from 0 to 5 were used: 0, normal interstitium; 1, <10% of areas injured; 2, 11–25% of areas injured; 3, 26–50% of areas injured; 4, 51–75% of areas injured; and 5, >76% of areas injured [61]. Mean values were calculated. Both scores (GSS and TIFS) were then added to produce a total score out of 9 for each evaluated rat [79].

### 4.8. Immunohistochemistry

Immunohistochemistry was performed using primary antibodies against TGF-β1 (cat # 215715, Abcam, Cambridge, MA, USA), vimentin(D21H3) (cat #5741, Cell Signaling, Danvers, MA, USA), and fibronectin (cat # 32419, Abcam, Cambridge, MA, USA). Antigen retrieval was performed using EDTA buffer at pH 8 for TGF-β1 and fibronectin and using citrate at pH 6 for vimentin before commencing IHC staining. After washing in phosphate-buffered saline (PBS), the slides were treated with biotinylated secondary antibodies for 20 min and streptavidin–horse radish peroxidase (HRP) for 20 min. Signals for immunoreactivity were visualized with the 3,3′-diaminobenzidine (DAB) substrate. The percentage of the positively stained areas was semi-quantitatively assessed using a four-tier scoring system: 0, no reactivity or less than 25% staining; 1, 25–50% positive staining, weak reactivity (light-brown staining); 2, 50–75% positive staining (medium-brown staining); 3, more than 75% positive staining reactivity (dark-brown to black staining) [80].

### 4.9. Statistical Analysis

Data are presented as the mean ± standard error of the mean (SEM). Non-parametric analysis was conducted using the Mann–Whitney U test and the Kruskal–Wallis tests for group comparisons, followed by the Manny-Whitney U test for post hoc pair comparisons. All the tests were run at a probability significance level of *p* < 0.05. All data were analyzed using SPSS software (SPSS Inc., Chicago, IL, USA, version 20.0).

## 5. Conclusions

The current preclinical study showed a significant favorable effect of a 2-week treatment with the Janus kinase (JAK) inhibitor ruxolitinib on the renal function and proinflammatory biomarkers of diabetic kidney disease in a rat model of STZ-induced diabetes mellitus over 8 weeks. JAK inhibitors should be investigated for a longer period in animals to prove a sustained renoprotective effect. These results pave the way for the use of ruxolitinib as a potential novel monotherapy or in combination with RAAS inhibitors in the treatment of diabetic kidney disease.

## Data Availability

Data is contained within the article and supplementary material.

## Supplementary Materials

The following are available online at https://www.mdpi.com/article/10.3390/ph14070608/s1, Figure. S1: Protein Functional Analysis Nework of JAK2. The analysis was performed using STRING functional protein network database. Table S1: Annotations of predicted Functional Partner of JAK2.
