# Supplementary material for: Early Renoprotective Effect of Ruxolitinib in a Rat Model of Diabetic Nephropathy
Source: Pharmaceuticals (Basel). 2021 Jun 24;14(7):608. doi: 10.3390/ph14070608 (PMC8308627; doi:10.3390/ph14070608)
Supplement: Supplementary file 1 [file pharmaceuticals-14-00608-s001.zip › pharmaceuticals-1282495-supplementary.pdf]

Supplementary files:

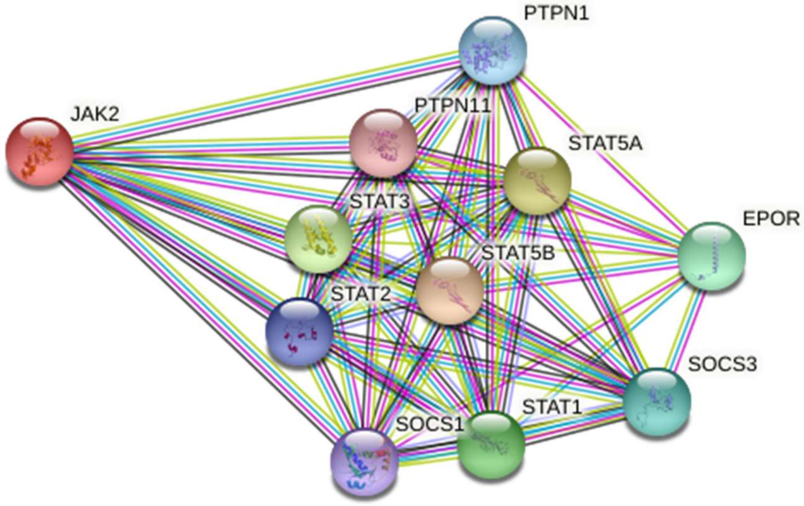

Supplementary Figure S1. Protein Functional Analysis Nework of JAK2. The analysis was performed using STRING functional protein network database

Supplementary Table S1. Annotations of predicted Functional Partner of JAK2

Protein	Annotation
STAT1	Signal transducer and activator of transcription 1-alpha/beta; Signal transducer and transcription activator that mediates cellular responses to interferons (IFNs), cytokine KITLG/SCF and other cytokines and other growth factors. Following type I IFN (IFN-alpha and IFN-beta) binding to cell surface receptors, signaling via protein kinases leads to activation of Jak kinases (TYK2 and JAK1) and to tyrosine phosphorylation of STAT1 and STAT2.
STAT2	Signal transducer and activator of transcription 2; Signal transducer and activator of transcription that mediates signaling by type I IFNs (IFN-alpha and IFN-beta). Following type I IFN binding to cell surface receptors, Jak kinases (TYK2 and JAK1) are activated, leading to tyrosine phosphorylation of STAT1 and STAT2.
STAT3	Signal transducer and activator of transcription 3; Signal transducer and transcription activator that mediates cellular responses to interleukins, and growth factors. Once activated, recruits coactivators, such as NCOA1 or MED1, to the promoter region of the target gene. May mediate cellular responses to activated FGFR1-4. Binds to the interleukin-6 (IL-6)-responsive elements identified in the promoters of various acute-phase protein genes. Activated by IL31 through IL31RA, and mediates the inflammatory response.
STAT5A	Signal transducer and activator of transcription 5A; it carries out a dual function: signal transduction and activation of transcription. It mediates cellular responses to growth factors including cytokine KITLG/SCF. It may mediate cellular responses to activated FGFR1-4. It binds to the GAS element and activates PRL-induced transcription and regulates the expression of milk proteins during lactation.
STAT5B	Signal transducer and activator of transcription 5B; it carries out a dual function: signal transduction and activation of transcription. It mediates cellular responses to the cytokine KITLG/SCF and other growth factors. Binds to the GAS element and activates PRL-induced transcription. Positively regulates hematopoietic/erythroid differentiation
SOCS1	Suppressor of cytokine signaling 1; SOCS family proteins form part of a classical negative feedback system that regulates cytokine signal transduction. SOCS1 is involved in negative regulation of cytokines that signal through the JAK/STAT3 pathway. Through binding to JAKs, inhibits their kinase activity. It is likely to be a major regulator of signaling by interleukin 6 (IL6) and leukemia inhibitory factor (LIF).
SOCS3	Suppressor of cytokine signaling 3; SOCS family proteins form part of a classical negative feedback system that regulates cytokine signal transduction. SOCS3 is involved in negative regulation of cytokines that signal through the JAK/STAT pathway. Inhibits cytokine signal transduction by binding to tyrosine kinase receptors. It binds to JAK2 and inhibits its kinase activity.
PTPN1	Tyrosine-protein phosphatase non-receptor type 1; Tyrosine-protein phosphatase which acts as a regulator of endoplasmic reticulum unfolded protein response. It mediates dephosphorylation of EIF2AK3/PERK; inactivating the protein kinase activity of EIF2AK3/PERK.
PTPN11	Tyrosine-protein phosphatase non-receptor type 11; Acts downstream of various receptor and cytoplasmic protein tyrosine kinases to participate in the signal transduction from the cell surface to the nucleus. Positively regulates MAPK signal transduction pathway.
EPOR	Erythropoietin receptor. It mediates erythropoietin- induced erythroblast proliferation and differentiation. Upon EPO stimulation, EPOR dimerizes triggering the JAK2/STAT5 signaling cascade.
